# Onco-miR-24 regulates cell growth and apoptosis by targeting BCL2L11 in gastric cancer

**DOI:** 10.1007/s13238-015-0234-5

**Published:** 2016-01-12

**Authors:** Haiyang Zhang, Jingjing Duan, Yanjun Qu, Ting Deng, Rui Liu, Le Zhang, Ming Bai, Jialu Li, Tao Ning, Shaohua Ge, Xia Wang, Zhenzhen Wang, Qian Fan, Hongli Li, Guoguang Ying, Dingzhi Huang, Yi Ba

**Affiliations:** Tianjin Medical University Cancer Institute and Hospital, Key Laboratory of Cancer Prevention and Therapy, National Clinical Research Center for Cancer, Tianjin, 300060 China; Department of Gastroenterology, Tianjin First Center Hospital, Tianjin, 300192 China; Changchun GeneScience Pharmaceuticals Co., Ltd, Changchun, 130012 China

**Keywords:** gastric cancer, BCL2L11, miR-24, tumorigenesis, cell apoptosis, proliferation, migration

## Abstract

Gastric cancer is one of the most common malignancies worldwide; however, the molecular mechanism in tumorigenesis still needs exploration. BCL2L11 belongs to the BCL-2 family, and acts as a central regulator of the intrinsic apoptotic cascade and mediates cell apoptosis. Although miRNAs have been reported to be involved in each stage of cancer development, the role of miR-24 in GC has not been reported yet. In the present study, miR-24 was found to be up-regulated while the expression of BCL2L11 was inhibited in tumor tissues of GC. Studies from both *in vitro* and *in vivo* shown that miR-24 regulates BCL2L11 expression by directly binding with 3′UTR of mRNA, thus promoting cell growth, migration while inhibiting cell apoptosis. Therefore, miR-24 is a novel onco-miRNA that can be potential drug targets for future clinical use.

## INTRODUCTION

Gastric cancer (GC) is one of the leading causes of cancer-related deaths all over the world (Fujita, 2009; Hartgrink et al., 2009). The conventional therapies for gastric cancer, such as chemotherapy and radiation, often lead to nonspecifically mass cell killing and side effects. Targeted medicine based on cancer gene therapy is a promising strategy for the treatment of gastric cancer (Hohenberger and Gretschel, 2003; Xiong et al., 2003). However, the number of effective targeted drugs for GC treatment is relatively less than that in other cancers. Studies on novel pathways of GC tumorigenesis provide candidates for drug target.

BCL2L11 is a member of BCL-2 family and is located in the outer membrane of mitochondria, where this protein acts as an important regulator that mediates excitotoxic apoptosis, apoptosis inducing factor translocation and mitochondrial depolarization (Concannon et al., 2010; Kilbride et al., 2010). Recent studies showed that BCL2L11 is a dual-agent that regulates autophagy in drug resistance (Dai and Grant, 2015), and BCL2L11 is also involved in neural apoptosis in methylmalonic academia (Li et al., 2014). The anti-apoptotic BCL2 members have multiple domains; while the pro-apoptotic members of BCL2 family, including BCL2L11, are BH3-domain-only proteins (Mansha et al., 2013; Frank et al., 2015; Huang et al., 2015). Although the role of BCL2L11 has been reported in these biological processes, the information that BCL2L11 regulates cell growth and apoptosis in cancer, especially in gastrointestinal tumors, remains largely unknown.

MicroRNAs (miRNAs) are a novel class of small non-coding RNAs (~22 bp) that are believed to regulate gene expression by directly binding with 3′UTR of target mRNAs, causing translational inhibition or mRNA degradation (Ambros, 2004; Bartel, 2004). MiRNAs are usually highly-conserved between species, and are regarded as distinct post-transcriptional regulators that are involved in most of the physiological and pathological processes, including cell differentiation, cell proliferation, the immune response, metabolism, aging process, tumorigenesis and drug resistance (Lim et al., 2005; Zhu et al., 2013; Zhang et al., 2015). Recent years, serum miRNAs are treated as a novel biomarker for the diagnosis and prognosis of various cancers (Liu et al., 2011; Liu et al., 2012; Ge et al., 2013; Luo et al., 2013; Wang et al., 2015). Recent studies by our group and others show that miRNAs can be secreted into the extracellular environment through microvesicles (MVs) and function as secretory signaling molecules that influence the recipient cell phenotypes (Zhang et al., 2010; Li et al., 2013; Li et al., 2015).

The expression of miR-24 has been found to be dys-regulated in several cancers (Lin et al., 2010; Qin et al., 2010; Naito et al., 2015; Organista-Nava et al., 2015; Zhao et al., 2015). Up-regulation of miR-24 in non-small cell lung cancer was found to promote cell proliferation (Zhao et al., 2015). It has been reported that miR-24 is also significantly up-regulated in gastric cancer (Volinia et al., 2006). However, the molecular mechanism that miR-24 regulate tumorigenesis in GC remains largely unknown. In the present study, we showed that miR-24 is up-regulated, while BCL2L11 expression is significantly suppressed in GC tissues. The subsequent luciferase assay showed that BCL2L11 is a direct target of miR-24; overexpression of miR-24 in GC cells leads to the inhibition of BCL2L11, thus promoting cell proliferation, migration while reducing cell apoptosis. *In vivo* experiments demonstrated that high level of miR-24 clearly accelerates while BCL2L11 overexpression strongly inhibits tumor growth. Therefore, our data illustrated a novel pathway comprising miR-24 and BCL2L11 in GC, which is a potential target for future clinical use.

## RESULTS

### BCL2L11 is down-regulated in gastric cancer

**Figure 1:**
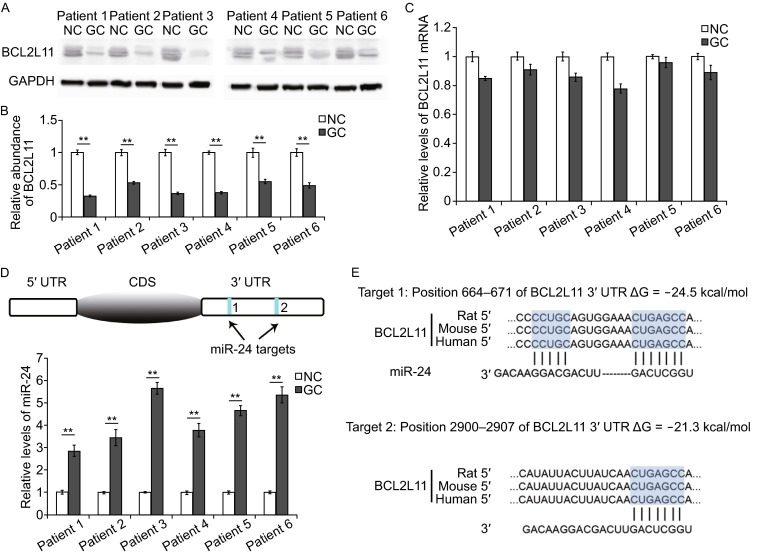
Inverse correlation between BCL2L11 and miR-24 in human GC tissues. (A) Western blot analysis of BCL2L11 expression in GC cancer tissues and the paired para-carcinoma tissues (*n* = 6). (B) Quantitative analysis of (A). (C) Relative levels of BCL2L11 mRNA levels in GC tissues (*n* = 6). (D and E) The predicted binding sites of miR-24 in the mRNA of BCL2L11 (D) and the base-pairing interaction between miR-24 and BCL2L11 mRNA (E). (F) Relative levels of miR-24 in GC tissues and para-carcinoma tissues (*n* = 6). ** indicates *P* < 0.01

Although BCL2L11 is well known to mediate cell apoptosis (Hagenbuchner et al., 2012; Luo and Rubinsztein, 2013; Dai and Grant, 2015), its expression pattern and the biological role in cancer have not been detailedly described yet. In this study, we first compared the mRNA levels and protein levels in gastric cancer tissues and the paired para-carcinoma tissues. The expression of BCL2L11 protein showed clear decrease in GC, which is reduced by nearly 70% of that in para-carcinoma tissues (Fig. 1A and 1B); however, its mRNA levels did not differ significantly between the cancer and noncancerous tissues (Fig. 1C). The disparity between mRNA and protein suggested that BCL2L11 expression mainly depends on post-transcriptional regulators.

### Identification of miR-24 as a potential upstream regulator of BCL2L11

One of the important modes of post-transcriptional regulation is miRNA-mediated repression of mRNA transcripts. By using bioinformatics tools, we found that miR-24 can directly target the 3′UTR of BCL2L11 mRNA (Fig. 1D). As is shown in Fig. 1E, miR-24 binds with BCL2L11 mRNA by complementary base pairing of two target regions.

It has been reported that miR-24 is significantly up-regulated in GC (Volinia et al., 2006), and is even higher after high-dose expose to radiation (Naito et al., 2015). We here valued the expression pattern of miR-24 in 6 pairs of tumor and cancer adjacent tissues. As is expected, miR-24 showed obvious increase in all the tumor tissues (Fig. 1F). Therefore, miR-24 is most likely to be the important regulator of BCL2L11 in gastric cancer cells.

### Validation of BCL2L11 as a direct target of miR-24

**Figure 2:**
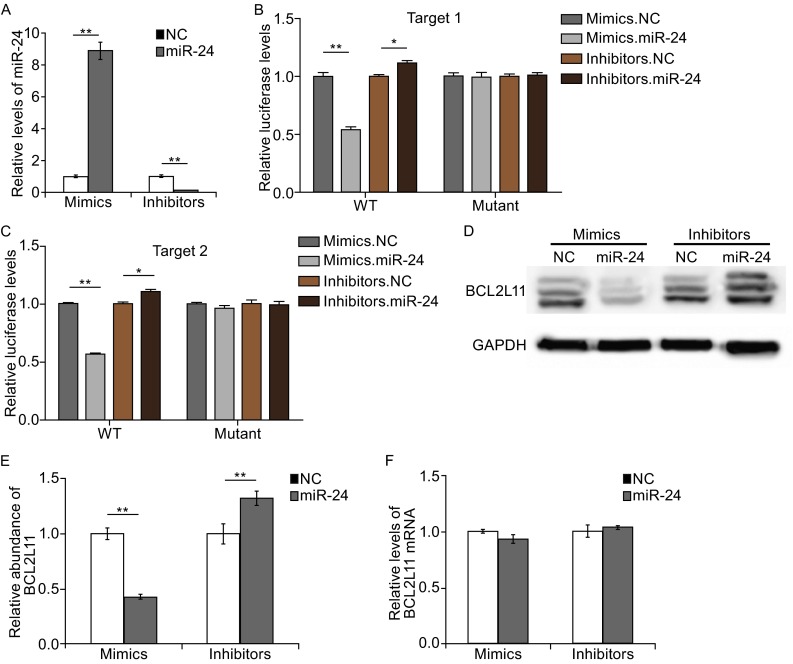
MiR-24 regulates BCL2L11 expression in gastric cancer cells. (A) Quantitative RT-PCR analysis of miR-24 levels in SGC7901 cells transfected with mimics or inhibitors. (B and C) Direct recognition of BCL2L11 by miR-24. HEK293T cells were co-transfected with firefly luciferase reporters containing either WT or mutant BCL2L11 3′UTR with miR-24 mimics and inhibitors. The interaction between miR-24 and target 1 (B) or target 2 (C) was shown respectively. (D) The suppression of BCL2L11 expression by miR-24 in SGC7901 cells. (E) Quantitative analysis of (D). (F) Quantitative RT-PCR analysis of BCL2L11 mRNA expression in SGC7901 cells. ** indicates *P* < 0.01; * indicates *P* < 0.05

The levels of miR-24 and BCL2L11 showed inverse correlation in GC, and the prediction by bioinformatics suggested that BCL2L11 is a potential target of miR-24; however, the direct evidence of the interaction between miR-24 and BCL2L11 given by luciferase assay is still needed. The relative luciferase activity was significantly inhibited by the co-transfection of miR-24 mimics and the luciferase reporters containing the predicted target regions of BCL2L11 mRNA (Fig. 2B and 2C); while the inhibition was lost when the binding sites in 3′UTR were mutated (Fig. 2B and 2C). The luciferase signal showed relative increase when miR-24 inhibitors were used instead (Fig. 2B and 2C).

The expressions of BCL2L11 protein and mRNA were also determined respectively after the overexpression or knockdown of miR-24 in SGC7901 cells. Relative levels of miR-24 in SGC7901 cells were also detected using qRT-PCR analysis following transfection of mimics or inhibitors (Fig. 1A). As is shown in Fig. 2D and 2E, the overexpression of miR-24 by transfection of mimics leads to the clear suppression of BCL2L11 protein, but not BCL2L11 mRNA. While the transfection of miR-24 inhibitors enhances the expression of BCL2L11 in SGC7901 cells (Fig. 2D and 2E). Meanwhile, BCL2L11 mRNA was not changed with the transfection of mimics or inhibitors (Fig. 2F).

These data demonstrated that miR-24 is an important regulator of BCL2L11 in GC cells, and miR-24 regulates BCL2L11 expression by directly targeting the 3′UTR of BCL2L11 mRNA.

### MiR-24 regulates proliferation, migration, apoptosis of SGC7901 cells

MiR-24 is also found to be obviously up-regulated in gastric cancer cell lines, SGC7901 and BGC823, compared with normal gastric cell line (GES-1) (Tchernitsa et al., 2010; Tsukamoto et al., 2010). In the present study, we assessed cell growth, cell metastasis and cell apoptosis in SGC7901 cells transfected with miR-24 mimics or inhibitors.

**Figure 3:**
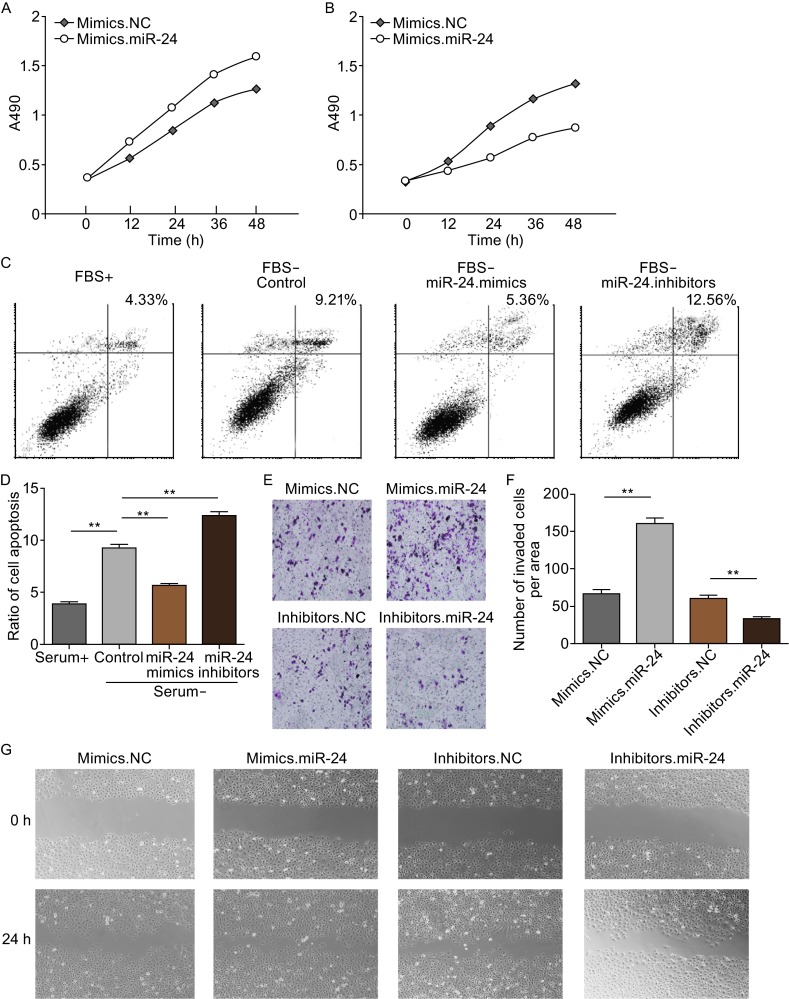
Onco-miR-24 regulates proliferation, apoptosis and migration of GC cells. SGC7901 cells were transfected with miR-24 mimics or inhibitors, and cell growth, apoptosis and migration were evaluated respectively. (A) Overexpression of miR-24 promotes cell proliferation of SGC7901 cells. (B) Knockdown of miR-24 suppress GC cell growth. (C) MiR-24 inhibits cell apoptosis of SGC7901 cells. (D) Quantitative analysis of (C). (E and F) Transwell assays show that miR-24 promotes cell migration of GC cells. (G) Validation of miR-24-mediated cell migration by scraping line method. ** indicates *P* < 0.01

CCK8 kit was used to measure the growth rate of SGC7901 cells. As is shown in Fig. 3A, high levels of miR-24 result in a clear increase in cell growth rate, while the inhibition of miR-24 leads to the significant suppression of cell proliferation (Fig. 3B).

The miR-24-BCL2L11 mediated cell apoptosis was assessed using cell flow assays. SGC7901 cells were transfected with miR-24 mimics and inhibitors, and fresh FBS-free RPMI-1640 medium was added to promote cell apoptosis. The remove of FBS caused a sharp increase of cell apoptosis compared with control; overexpression of miR-24 reduced the ratio of cell apoptosis, while transfection of miR-24 inhibitor further promoted cell apoptosis (Fig. 3C and 3D).

Effects of miR-24 on cell migration were valued by transwell assay and scrapping method. SGC7901 cells transfected with miR-24 mimics showed a higher ratio in migration (Fig. 3E–G). However, cell migration was strongly inhibited when cells are transfected with miR-24 inhibitors (Fig. 3E–G).

These results based on *in vitro* experiments demonstrated that miR-24 is an onco-miRNA in gastric cancer, and plays a key role in the biological processes in cancer cells.

### Overexpression of BCL2L11 partly reduced miR-24-induced GC cell growth

**Figure 4:**
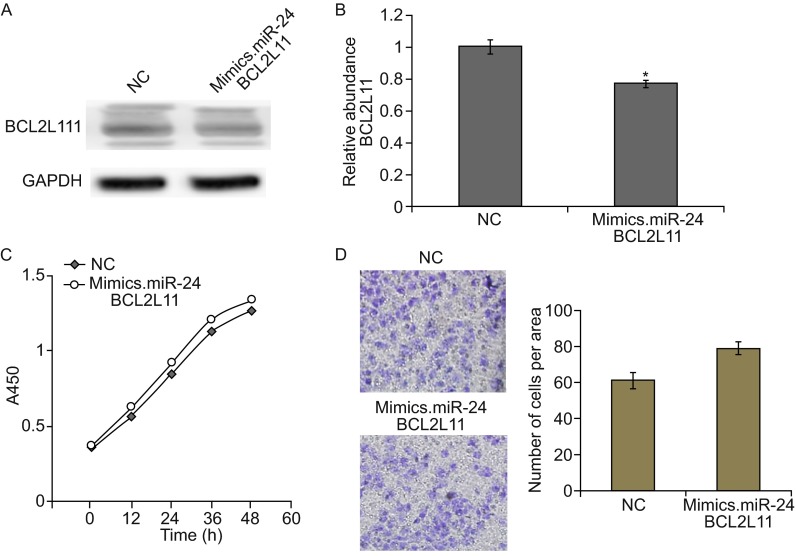
BCL2L11 rescues miR-24-induced cell growth and migration. SGC7901 cells were co-transfected with miR-24 mimics and BCL2L11 lenti-virus plasmid. The protein levels were detected by Western blot, and cell growth and migration were determined by CCK8 and Transwell assays respectively. (A) Western blot analysis of BCL2L11 expression in SGC7901 cells co-overexpressed with miR-24 and BCL2L11. (B) Quantitative analysis of (A). (C and D). Cell proliferation (C) and migration (D) of SGC7901 cells treated with miR-24 mimics and BCL2L11 lenti-virus simultaneously. ** indicates *P* < 0.01

To give more evidence that miR-24 promotes tumorigenesis in gastric cancer, we used miR-24 mimics and lenti-virus particles to overexpress miR-24 and BCL2L11 in SGC7901 cells simultaneously. As is shown in Fig. 4A and 4B, co-transfection of miR-24 mimics and BCL2L11 lenti-virus particles partly rescued BCL2L11 expression compared with the single transfection of miR-24 mimics (Fig. 2D). The overexpression of BCL2L11 in SGC7901 cells partly decreased miR-24-induced cell proliferation (Fig. 4C) and migration (Fig. 4D).

### Effects of BCL2L11 silencing in SGC7901

**Figure 5:**
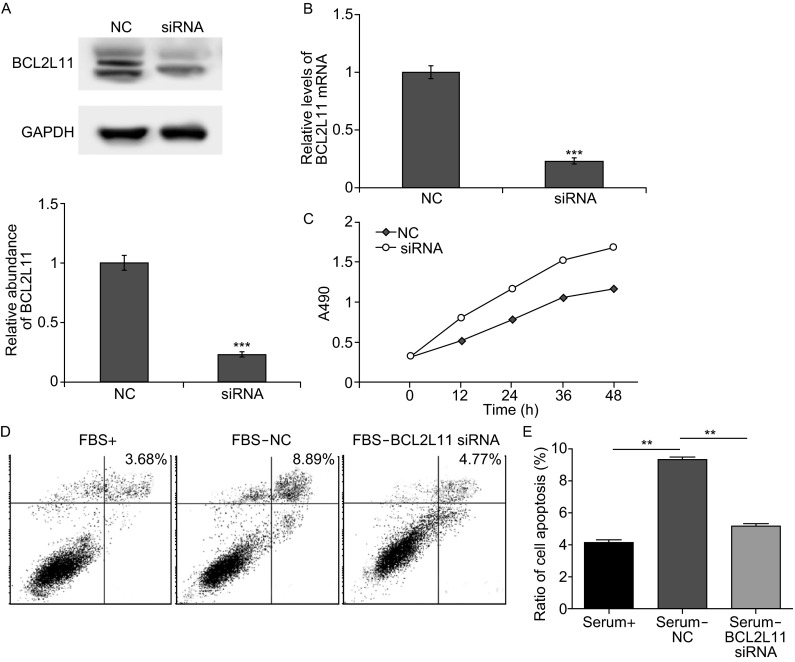
Knock-down of BCL2L11 in SGC7901 cells. (A and B) Silencing of BCL2L11 expression by siRNA. SGC7901 cells were transfected with BCL2L11 siRNA, and the protein levels (A) and mRNA levels (B) were detected respectively. (C) Knock-down of BCL2L11 strongly enhances cell growth in GC cells. (D and E) Silencing of BCL2L11 clearly reduces cell apoptosis of SGC7901 cells. ** indicates *P* < 0.01; *** indicated *P* < 0.001

To give a better understanding of BCL2L11-involved pathway in gastric cancer cells, siRNA was used to knock-down BCL2L11 in SGC7901 cells. Transfection of BCL2L11 siRNA leads to a sharp decrease in protein expression (Fig. 5A and 5B), thus significantly suppressing cell apoptosis and promoting cell proliferation (Fig. 5C–E). Therefore, BCL2L11 acts as a cancer suppresser in GC; its dramatic down-regulation contributes to the reduced cell death and fast cell growth rate in cancer.

### The miR-24-BCL2L11 pathway regulates tumor growth *in vivo*

**Figure 6:**
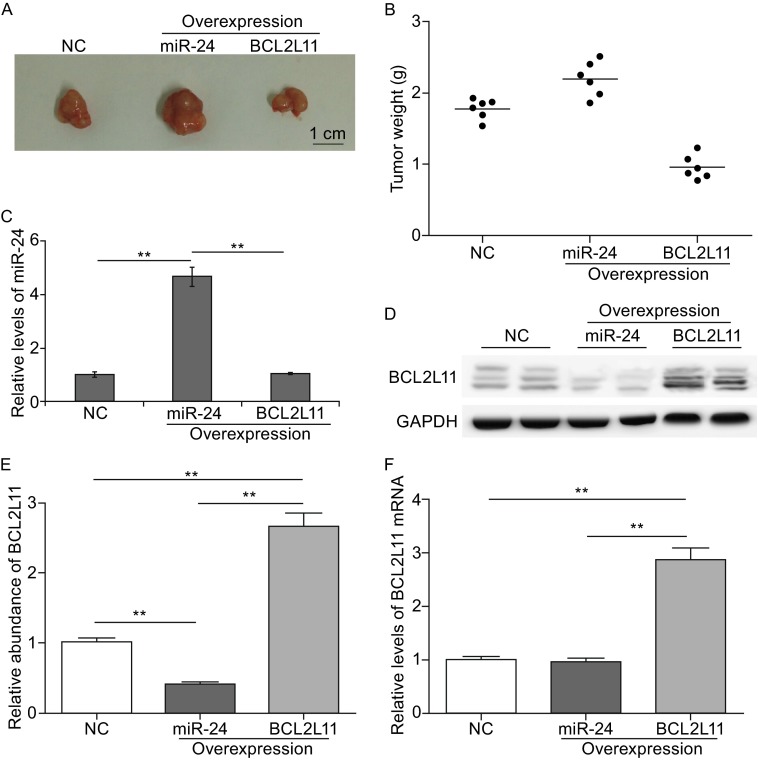
MiR-24 promotes tumorigenesis by suppressing BCL2L11 expression *in vivo*. (A) Representative image of tumors excised from nude mice. SGC7901 cells were treated with miR-24-overexpressing lenti-virus, BCL2L11-overexpressing lenti-virus and the control lenti-virus respectively, and cells were implanted in BALB/c mice. (B) Weight of tumors from mice implanted with SGC7901 cells. (C) Quantitative RT-PCR analysis of miR-24 in implanted tumors. (D–F) BCL2L11 expression in implanted tumors. Western blot analysis of BCL2L11 levels (D); quantitative analysis of (E); Relative levels of BCL2L11 mRNA in implanted tumors (F). ** indicates *P* < 0.01

Subsequently, we assessed the effects of miR-24 or BCL2L11 in tumor growth by using mouse implanted tumor model. SGC7901 cells were transfected with lentivirus particles to overexpress miR-24 or BCL2L11 respectively, and cells were injected subcutaneously in the armpit of nude mice. As is shown in Fig. 6A and 6B, overexpression of miR-24 increases tumor size and weight obviously; while the overexpression of BCL2L11 strongly inhibits tumor growth. The levels of miR-24 and BCL2L11 were also detected in the excised tumors. It is showed that miR-24 was elevated fivefold in miR-24-overexpression group, and BCL2L11 increased nearly 3 folds in BCL2L11-overexpression group compared with control (Fig. 6C–E). These results based on *in vivo* experiments further demonstrated that the miR-24 acts as an onco-miRNA by inhibiting BCL2L11 expression in gastric cancer.

## DISCUSSION

Given the current incomplete knowledge of the pathways comprising of miRNAs and the down-stream target proteins in cancer, more studies are needed to be focused on these dys-regulated miRNAs. MiRNA profiles are believed to provide an accurate diagnosis for different types of cancer, contributing to the disorder of protein expression in cancer cells. Although miR-24 has been found to be up-regulated in both GC tissues and cell lines (Volinia et al., 2006; Tchernitsa et al., 2010; Tsukamoto et al., 2010), its function in the process of tumorigenesis remains unclear. Moreover, the expression pattern and function of BCL2L11 in cancer, especially in GC, have not been explored yet.

In this study, miR-24 and BCL2L11 levels showed opposite trends in 6 pairs of gastric carcinoma and para-carcinoma tissues. The obvious inhibition of BCL2L11 is surely to contribute to decreased apoptosis and accelerated proliferation of cancer cells, and is resulted from the high expression of miR-24. The subsequent experiments suggest that the miR-24-BCL2L11 pathway is involved in the processes of cell growth, migration and apoptosis, thus regulating tumorigenesis. We have previously reported the serum miRNA profile in GC, and found that miR-24 is down-regulated in the serum of GC patients (Liu et al., 2011). We believed that the secretion of onco-miR-24 from gastric cancer cells is reduced, resulting in the up-regulation of miR-24 in cancer cells and the down-regulation in serum. However, this needs more evidence in further studies.

For decades, chemotherapy and radiation are the main therapeutic methods for cancer, causing cell killing without specific targeting (Amit et al., 2011; McBride et al., 2013; Manyam et al., 2015). A better comprehension of cancer, especially the molecular mechanism that regulates tumor growth, provides novel drug targets for future clinical use. MiRNAs are proved to be a useful biomarker in the diagnosis and prognosis of cancer, new diagnostic kit based on serum miRNA profile have been developed for consumer marketing (Lin et al., 2015). Our group had reported that miRNAs and other small RNAs trafficked by MVs or plasmids can be used for the treatment of diseases, including cancer, in mouse models (Liu et al., 2013; Yin et al., 2014; Zhang et al., 2014). Therefore, miRNAs have a good prospect in clinical application.

To conclude, we illustrated that an important cell-apoptosis initiator, BCL2L11, is regulated by miR-24 in gastric cancer. This novel pathway provides potential targets by using miRNAs for gene therapy.

## MATERIALS AND METHODS

### Animals

Male nude mice (BALB/c-nu, 6~8 weeks) were purchased and were housed in a pathogen-free animal facility with access to water and food and allowed to eat and drink ad libitum. All of the experimental procedures were performed in accordance with protocols approved by the Institutional Animal Care and Research Advisory Committee of Tianjin Medical University.

### Human tissue

Human gastric cancer tissues and paired adjacent noncancerous tissues were derived from patients undergoing a surgical procedure at the Tianjin Medical University Cancer Institute and Hospital (Tianjin, China). Both tumor tissues and noncancerous tissues were confirmed histologically. The pathological type of each cancer was determined to be adenocarcinoma. Written consents were provided by all of the patients, and the Ethics Committee of Tianjin Medical University Cancer Institute and Hospital approved all aspects of this study. Tissue fragments were immediately frozen in liquid nitrogen at the time of surgery and stored at −80°C.

### Cell culture

Human gastric cell line SGC7901, human embryo kidney epithelial cell line HEK293T were cultured in DMEM (Gibco, USA), which was supplemented with 10% fetal bovine serum (FBS, Gibco, USA) in a humidified incubator at 37°C with 5% CO_2_.

### RNA isolation and quantitative RT-PCR

Assays to quantify mature miRNAs were conducted as previously described with slight modifications (Chen et al., 2005; Andrews et al., 2015). Total RNA was extracted from the cultured cells and tissues using TRIzol Reagent (Invitrogen) according to the manufacturer’s instructions. miRNA determination was performed using Taqman microRNA probes (Applied Biosystems, Foster City, CA). All of the reactions were run in triplicate. After the reactions were completed, the cycle threshold (C_T_) data were determined using fixed threshold settings, and the mean C_T_ was determined from triplicate PCRs. A comparative C_T_ method was used to compare each condition to the control reactions. U6 snRNA was used as an internal control of miRNAs, and the mRNA levels of BCL2L11 was normalized to GAPDH. The relative amount of gene normalized to control was calculated with the equation 2^−ΔCT^, in which ΔC_T_ = C_T__gene_ − C_T_ control. Primers of BCL2L11 and GAPDH were as follows: 5′-AGAAGGCTGGGGCTCATTTG-3′ (GAPDH, sense); 5′-AGGGGCCATCCACAGTCTTC-3′ (GAPDH, antisense); 5′-CACCAGCACCATAGAAGAA-3′ (BCL2L11, sense); 5′-ATAAGGAGCAGGCACAGA-3′ (BCL2L11, antisense).

### Cell transfection

SGC 7901 Cells were seeded in a 6-well plate and transfection was conducted after 24 h. The BCL2L11 overexpressing lentivirus and the control lentivirus were bought from GenePharma (Shanghai, China),and 10^6^ lentivirus were added into every single well with gentle mixing. Cell transfection with miR-24 mimics and inhibitors were conducted by using Lipofectamine 2000 (Invitrogen) according to the manufacturer’s instructions. For each well, equal doses (100 pmol) of miRNA mimics, inhibitors, siRNAs (Santa Cruz, sc-29802), or scrambled negative control RNA were used. The cells were harvested at 24 h after transfection for real-time quantitative PCR analysis and western blotting.

### Luciferase assay

Part of the wild type and mutated 3′UTR of BCL2L11, containing the predicted miR-24 targeting regions, was synthesized and inserted into a p-MIR-report plasmid (Genescript, Nanjing, China). For luciferase reporter assays, 2 mg of firefly luciferase reporter plasmid, 2 mg of β-galactosidase expression vector (Ambion), and equal amounts (200 pmol) of mimics, inhibitors, or scrambled negative control RNA were transfected into cells. And the β-galactosidase vector was used as a transfection control. At 24 h after transfection, cells were assayed using luciferase assay kit (Promega).

### Cell flow assays

For cell cycle analysis, cells were washed with phosphate-buffered saline solution (PBS) and fixed in 70% ethanol at 4°C for 2–4 h. After fixation, cells were washed twice with PBS before re-suspension in propidium iodide/RNase A solution (5 μg/mL propidium iodide and 100 mg/mL RNase A). Cells were incubated with propidium iodide at room temperature in the dark for 1 h, then the stained cells were analyzed by flow cytometry.

For cell apoptosis analysis, cells were cultured overnight with both serum-containing complete medium and serum-depleted medium; the attached cells and floating cells were then harvested. Flow cytometry analysis of apoptotic cells was carried out using an Annexin V-FITC/PI staining kit (BD Biosciences, CA, USA). After washes with cold PBS, the cells were re-suspended in binding buffer (100 mmol/L HEPES, pH 7.4, 100 mmol/L NaCl, and 25 mmol/L CaCl_2_) followed by staining with Annexin V-FITC/PI at room temperature in darkness for 15 min. Apoptotic cells were then evaluated by gating PI and Annexin V-positive cells on a fluorescence-activated cell-sorting (FACS) flow cytometer (BD Biosciences, San Jose, CA). All experiments were performed in triplicate.

### Cell proliferation assay

SGC7901 cells were collected at 12 h, 24 h, 36 h and 48 h post-transfection; 10 μL of WST-8 was added into a corresponding test well and incubated for 4 h. Absorbance was measured at a wavelength of 490 nm.

### Cell migration assay

Migration assays were performed using either scrapping line method or 6.5 mm Transwell^®^ with 8.0 μm Pore Polycarbonate Membrane Insert (Corning, New York, USA) according to the manufacturer’s instructions. At 24 h post-transfection with miR-24 mimics, inhibitors and controls, SGC-7901 cells were incubated in for 24 h, and then 1 × 10^5^ cells in 200 μL serum-free medium were added to the upper chamber. A volume of 500 μL of 10% FBS-containing medium was then added to the lower chamber as a chemo-attractant. Cells were incubated for another 24 h at 37°C, and then non-migrating cells on the upper surface of the membrane were gently scraped off with cotton swabs. Cells that migrated to the bottom of the membrane were stained with the cell stain provided in the assay kit for 20 min and visualized under a microscope. To minimize the bias, at least three randomly selected fields with 200× magnification were counted, and the average number was taken.

### Establishment of tumor xenograft in nude mice

SGC7901 cells treated with control lentivirus or miR-24 overexpressing lentivirus or BCL2L11 overexpressing lentivirus were injected subcutaneously into nude mice (1 × 10^7^ cells for one mouse). Mice were sacrificed after 4 weeks, and the weight and diameter of tumors were recorded.

### The miRNA target prediction

The miRNA target prediction and analysis were performed with the algorithms from TargetScan (http://www.targetscan.org/) PicTar (http://pictar.mdc-berlin.de/) and miRanda (http://www.microrna.org/).

### Western blotting analysis

The BCL2L11 expression was assessed by Western blotting analysis and samples were normalized to GAPDH. Protein extraction was blocked with PBS-5% fat-free dried milk at room temperature for 1 h and incubated at 4°C overnight with anti-BCL2L11 (1:500, Santa Cruz), and anti-GAPDH (1:2000, Santa Cruz) antibodies respectively.

### Statistical analyses

All data were representative of at least three independent experiments. Data were expressed as mean ± S.E.M of at least three separate experiments. Statistical significance was considered at *P* < 0.05 using the Student’s *t*-test. In this study, ‘*’ indicates ‘*P* < 0.05’, ‘**’ indicates ‘*P* < 0.01’, and ‘***’ indicates ‘*P* < 0.001’.

## ABBREVIATIONS

C_T_: cycle threshold
FACS: fluorescence-activated cell-sorting
GC: gastric cancer
miRNAs: microRNAs
MVs: microvesicles

## References

[CR1] Ambros V (2004). The functions of animal microRNAs. Nature.

[CR2] Amit D, Tamir S, Birman T, Gofrit ON, Hochberg A (2011). Development of targeted therapy for bladder cancer mediated by a double promoter plasmid expressing diphtheria toxin under the control of IGF2-P3 and IGF2-P4 regulatory sequences. Int J Clin Exp Med.

[CR3] Andrews WJ, Brown ED, Dellett M, Hogg RE, Simpson DA (2015). Rapid quantification of microRNAs in plasma using a fast real-time PCR system. Biotechniques.

[CR4] Bartel DP (2004). MicroRNAs: genomics, biogenesis, mechanism, and function. Cell.

[CR5] Chen C, Ridzon DA, Broomer AJ, Zhou Z, Lee DH, Nguyen JT, Barbisin M, Xu NL, Mahuvakar VR, Andersen MR (2005). Real-time quantification of microRNAs by stem-loop RT-PCR. Nucleic Acids Res.

[CR6] Concannon CG, Tuffy LP, Weisova P, Bonner HP, Davila D, Bonner C, Devocelle MC, Strasser A, Ward MW, Prehn JH (2010). AMP kinase-mediated activation of the BH3-only protein Bim couples energy depletion to stress-induced apoptosis. J Cell Biol.

[CR7] Dai Y, Grant S (2015). BCL2L11/Bim as a dual-agent regulating autophagy and apoptosis in drug resistance. Autophagy.

[CR8] Frank DO, Dengjel J, Wilfling F, Kozjak-Pavlovic V, Hacker G, Weber A (2015). The pro-apoptotic BH3-only protein Bim interacts with components of the translocase of the outer mitochondrial membrane (TOM). PLoS One.

[CR9] Fujita T (2009) Gastric cancer. Lancet, 374:1593–1594; author reply 1594–1595 10.1016/S0140-6736(09)61947-4 19897123

[CR10] Ge Y, Zhao K, Qi Y, Min X, Shi Z, Qi X, Shan Y, Cui L, Zhou M, Wang Y (2013). Serum microRNA expression profile as a biomarker for the diagnosis of pertussis. Mol Biol Rep.

[CR11] Hagenbuchner J, Kuznetsov A, Hermann M, Hausott B, Obexer P, Ausserlechner MJ (2012). FOXO3-induced reactive oxygen species are regulated by BCL2L11 (Bim) and SESN3. J Cell Sci.

[CR12] Hartgrink HH, Jansen EP, van Grieken NC, van de Velde CJ (2009). Gastric cancer. Lancet.

[CR13] Hohenberger P, Gretschel S (2003). Gastric cancer. Lancet.

[CR14] Huang C, Li J, Hong K, Xia Z, Xu Y, Cheng X (2015). BH3-only protein Bim is upregulated and mediates the apoptosis of cardiomyocytes under glucose and oxygen-deprivation conditions. Cell Biol Int.

[CR15] Kilbride SM, Farrelly AM, Bonner C, Ward MW, Nyhan KC, Concannon CG, Wollheim CB, Byrne MM, Prehn JH (2010). AMP-activated protein kinase mediates apoptosis in response to bioenergetic stress through activation of the pro-apoptotic Bcl-2 homology domain-3-only protein BMF. J Biol Chem.

[CR16] Li J, Zhang Y, Liu Y, Dai X, Li W, Cai X, Yin Y, Wang Q, Xue Y, Wang C (2013). Microvesicle-mediated transfer of microRNA-150 from monocytes to endothelial cells promotes angiogenesis. J Biol Chem.

[CR17] Li Y, Peng T, Li L, Wang X, Duan R, Gao H, Guan W, Lu J, Teng J, Jia Y (2014). MicroRNA-9 regulates neural apoptosis in methylmalonic acidemia via targeting BCL2L11. Int J Dev Neurosci.

[CR18] Li J, Zhang Y, Li D, Liu Y, Chu D, Jiang X, Hou D, Zen K, Zhang CY (2015). Small non-coding RNAs transfer through mammalian placenta and directly regulate fetal gene expression. Protein Cell.

[CR19] Lim LP, Lau NC, Garrett-Engele P, Grimson A, Schelter JM, Castle J, Bartel DP, Linsley PS, Johnson JM (2005). Microarray analysis shows that some microRNAs downregulate large numbers of target mRNAs. Nature.

[CR20] Lin SC, Liu CJ, Lin JA, Chiang WF, Hung PS, Chang KW (2010). miR-24 up-regulation in oral carcinoma: positive association from clinical and in vitro analysis. Oral Oncol.

[CR21] Lin XJ, Chong Y, Guo ZW, Xie C, Yang XJ, Zhang Q, Li SP, Xiong Y, Yuan Y, Min J (2015). A serum microRNA classifier for early detection of hepatocellular carcinoma: a multicentre, retrospective, longitudinal biomarker identification study with a nested case-control study. Lancet Oncol.

[CR22] Liu R, Zhang C, Hu Z, Li G, Wang C, Yang C, Huang D, Chen X, Zhang H, Zhuang R (2011). A five-microRNA signature identified from genome-wide serum microRNA expression profiling serves as a fingerprint for gastric cancer diagnosis. Eur J Cancer.

[CR23] Liu R, Chen X, Du Y, Yao W, Shen L, Wang C, Hu Z, Zhuang R, Ning G, Zhang C (2012). Serum microRNA expression profile as a biomarker in the diagnosis and prognosis of pancreatic cancer. Clin Chem.

[CR24] Liu YC, Zhao LM, Li DM, Yin Y, Zhang CY, Li J, Zhang YJ (2013). Microvesicle-delivery miR-150 promotes tumorigenesis by up-regulating VEGF, and the neutralization of miR-150 attenuate tumor development. Protein Cell.

[CR25] Luo S, Rubinsztein DC (2013). BCL2L11/BIM: a novel molecular link between autophagy and apoptosis. Autophagy.

[CR26] Luo Y, Wang C, Chen X, Zhong T, Cai X, Chen S, Shi Y, Hu J, Guan X, Xia Z (2013). Increased serum and urinary microRNAs in children with idiopathic nephrotic syndrome. Clin Chem.

[CR27] Mansha M, Hussain A, Kofler A, Grubbauer C, Goetsch K, Ploner C, Kofler R (2013). “Bam,” a novel glucocorticoid-induced BH3-only transcript from the BCL2L11/Bim locus, does not appear to be translated. Leuk Lymphoma.

[CR28] Manyam BV, Mallick IH, Abdel-Wahab MM, Reddy CA, Remzi FH, Kalady MF, Lavery I, Koyfman SA (2015). The impact of preoperative radiation therapy on locoregional recurrence in patients with stage IV rectal cancer treated with definitive surgical resection and contemporary chemotherapy. J Gastrointest Surg.

[CR29] McBride SM, Raut CP, Lapidus M, Devlin PM, Marcus KJ, Bertagnolli M, George S, Baldini EH (2013). Locoregional recurrence after preoperative radiation therapy for retroperitoneal sarcoma: adverse impact of multifocal disease and potential implications of dose escalation. Ann Surg Oncol.

[CR30] Naito Y, Oue N, Pham TT, Yamamoto M, Fujihara M, Ishida T, Mukai S, Sentani K, Sakamoto N, Hida E (2015). Characteristic miR-24 expression in gastric cancers among atomic bomb survivors. Pathobiology.

[CR31] Organista-Nava J, Gomez-Gomez Y, Illades-Aguiar B, Del Carmen Alarcon-Romero L, Saavedra-Herrera MV, Rivera-Ramirez AB, Garzon-Barrientos VH, Leyva-Vazquez MA (2015). High miR-24 expression is associated with risk of relapse and poor survival in acute leukemia. Oncol Rep.

[CR32] Qin W, Shi Y, Zhao B, Yao C, Jin L, Ma J, Jin Y (2010). miR-24 regulates apoptosis by targeting the open reading frame (ORF) region of FAF1 in cancer cells. PLoS One.

[CR33] Tchernitsa O, Kasajima A, Schafer R, Kuban RJ, Ungethum U, Gyorffy B, Neumann U, Simon E, Weichert W, Ebert MP (2010). Systematic evaluation of the miRNA-ome and its downstream effects on mRNA expression identifies gastric cancer progression. J Pathol.

[CR34] Tsukamoto Y, Nakada C, Noguchi T, Tanigawa M, Nguyen LT, Uchida T, Hijiya N, Matsuura K, Fujioka T, Seto M (2010). MicroRNA-375 is downregulated in gastric carcinomas and regulates cell survival by targeting PDK1 and 14-3-3zeta. Cancer Res.

[CR35] Volinia S, Calin GA, Liu CG, Ambs S, Cimmino A, Petrocca F, Visone R, Iorio M, Roldo C, Ferracin M (2006). A microRNA expression signature of human solid tumors defines cancer gene targets. Proc Natl Acad Sci USA.

[CR36] Wang C, Hu J, Lu M, Gu H, Zhou X, Chen X, Zen K, Zhang CY, Zhang T, Ge J (2015). A panel of five serum miRNAs as a potential diagnostic tool for early-stage renal cell carcinoma. Sci Rep.

[CR37] Xiong HQ, Gunderson LL, Yao J, Ajani JA (2003). Chemoradiation for resectable gastric cancer. Lancet Oncol.

[CR38] Yin Y, Cai X, Chen X, Liang HW, Zhang YJ, Li J, Wang ZY, Chen XL, Zhang W, Yokoyama S (2014). Tumor-secreted miR-214 induces regulatory T cells: a major link between immune evasion and tumor growth. Cell Resh.

[CR39] Zhang Y, Liu D, Chen X, Li J, Li L, Bian Z, Sun F, Lu J, Yin Y, Cai X (2010). Secreted monocytic miR-150 enhances targeted endothelial cell migration. Mol Cell.

[CR40] Zhang YQ, Li LM, Yu JX, Zhu DH, Zhang YJ, Li XH, Gu HW, Zhang CY, Zen K (2014). Microvesicle-mediated delivery of transforming growth factor beta 1 siRNA for the suppression of tumor growth in mice. Biomaterials.

[CR41] Zhang H, Yang H, Zhang C, Jing Y, Wang C, Liu C, Zhang R, Wang J, Zhang J, Zen K (2015). Investigation of microRNA expression in human serum during the aging process. J Gerontol A Biol Sci Med Sci.

[CR42] Zhao G, Liu L, Zhao T, Jin S, Jiang S, Cao S, Han J, Xin Y, Dong Q, Liu X (2015). Upregulation of miR-24 promotes cell proliferation by targeting NAIF1 in non-small cell lung cancer. Tumour Biol.

[CR43] Zhu D, Pan C, Li L, Bian Z, Lv Z, Shi L, Zhang J, Li D, Gu H, Zhang CY (2013). MicroRNA-17/20a/106a modulate macrophage inflammatory responses through targeting signal-regulatory protein alpha. J Allergy Clin Immunol.

